# Is the NEI-VFQ-25 a useful tool in identifying visual impairment in an elderly population?

**DOI:** 10.1186/1471-2415-6-24

**Published:** 2006-06-09

**Authors:** Christopher G Owen, Alicja R Rudnicka, Liam Smeeth, Jennifer R Evans, Richard PL Wormald, Astrid E Fletcher

**Affiliations:** 1 Division of Community Health Sciences, St George's, University of London, Cranmer Terrace, London, SW17 0RE, UK; 2 Centre for Ageing and Public Health, London School of Hygiene and Tropical Medicine, Keppel Street, London, WC1E 7HT, UK; 3 Moorfields Eye Hospital, City Road, London, EC1V 2PD, UK

## Abstract

**Background:**

The use of self-report questionnaires to substitute for visual acuity measurement has been limited. We examined the association between visual impairment and self reported visual function in a population sample of older people in the UK.

**Methods:**

Cross sectional study of people aged more than 75 years who initially participated in a trial of health screening. The association between 25-item National Eye Institute Visual Function Questionnaire (NEI-VFQ) scores and visual impairment (defined as an acuity of less than 6/18 in the better eye) was examined using logistic regression.

**Results:**

Visual acuity and NEI-VFQ scores were obtained from 1807 participants (aged 77 to 101 years, 36% male), from 20 general practices throughout the UK. After adjustment for age, gender, practice and NEI-VFQ sub-scale scores, those complaining of poor vision in general were 4.77 times (95% CI 3.03 to 7.53) more likely to be visually impaired compared to those who did not report difficulty. Self-reported limitations with social functioning and dependency on others due to poor vision were also associated with visual impairment (odds ratios, 2.52, 95% CI 1.55 to 4.11; 1.73, 95% CI 1.05 to 2.86 respectively). Those reporting difficulties with near vision and colour vision were more likely to be visually impaired (odds ratios, 2.32, 95% CI 1.30 to 4.15; 2.25, 95% CI 1.35 to 3.73 respectively). Other NEI-VFQ sub-scale scores were unrelated to measures of acuity. Similar but weaker odds ratios were found with reduced visual acuity (defined as less than 6/12 in the better eye). Although differences in NEI-VFQ scores were small, scores were strongly associated with visual acuity, binocular status, and difference in acuity between eyes.

**Conclusion:**

NEI-VFQ questions regarding the quality of general vision, social functioning, visual dependency, near vision and colour vision are strongly and independently associated with an objective measure of visual impairment in an elderly population.

## Background

Visual acuity is a clinical measure of an individual's ability to perform specific visual tasks. Previous population-based studies have shown that reduced visual acuity, so called 'visual impairment', is related to an individual's everyday task performance and self-reported difficulty with everyday tasks related to vision [1-3]. The National Eye Institute Visual Function Questionnaire (NEI-VFQ) was developed to give a self-reported measure of visual function. A 51-item questionnaire was originally devised in the US from focus groups of people with major causes of eye disease [4]. The questionnaire was later shortened to 25 items, based predominantly on the responses from those with eye disease and visual impairment, and also from a minority group without eye disease [5]. The shortened questionnaire has been validated and used to show that those with ocular disease and accompanying visual impairment have lower scores compared to a reference group without ocular disease or visual impairment [6-8]. The use of self-report questionnaires to substitute for visual acuity measurement has been limited [9], although the NEI-VFQ has been used in adult populations (aged 40 years or more) to show that those with visual impairment have lower scores compared to those without reduced visual acuity [10,11]. However, concerns about the validity of certain sub-scales used in the NEI-VFQ and its range of measurement have been raised [12]. In addition, use of the NEI VFQ in non-US populations is limited, especially amongst older populations who are likely to experience higher levels of visual difficulties than younger age groups.

We have examined the association between responses to the NEI-VFQ and objective measures of visual impairment (based on established cut-offs in visual acuity). The extent of reported visual difficulties amongst an elderly population (aged over 75 years) in the UK is also described.

## Methods

The study design is a cross sectional study of those who initially participated in the Medical Research Council's (MRC) trial of assessment and management of older people in the community. Details of the trial have been described in detail elsewhere [13]. In brief, 106 general practices from the UK Medical Research Council General Practice Research Framework were recruited to the trial. The sampling of practices was stratified by tertiles of Standardised Mortality Ratio (mortality experience of local area relative to national mortality) and Jarman score (an area deprivation measure indicator) to ensure a representative sample. Practices were randomised to two groups: targeted versus universal screening. In the universal screening group, all participants were invited to have a detailed health assessment by a research nurse that included visual acuity. All patients aged 75 years or over registered with participating general practices were included in the study, unless they were resident in a long stay hospital or psychogeriatric care facility or were terminally ill. People in sheltered or residential housing for the elderly were included. The examinations took place between 1995 and 1999. Three to five years later, as part of a nested trial evaluating the benefit of vision screening [14], 4340 previously sampled participants from 20 general practices were selected at random. Of the 2875 who were still alive, 2589 (90%) were invited to have an assessment of their vision and complete the 25-item NEI-VFQ. Research nurses measured presenting monocular and binocular visual acuity (defined as using their habitual distance correction) on the logMAR scale using Glasgow acuity charts [15]. The 25 questions in the NEI-VFQ are grouped in 12 sub-scales (including general health, general vision, ocular pain, near activities, distance activities, social functioning, mental health, role difficulties, dependency, driving, colour vision and peripheral vision), as well as a combined total score (Table 1). Each sub-scale was calculated according to the methods described by the NEI-VFQ developers and can range from 0 to 100, where 0 is the worst and 100 represents no disability related to vision (that is, ratings of excellent or no difficulty). Demographic data including age, gender, and socioeconomic status were also collected. Socioeconomic status was measured using the Carstairs deprivation index, where low scores represent those from areas associated with less privileged circumstances [16].

**Table 1:** Questions used to derive NEI-VFQ sub-scale scores

NEI-VFQ sub-scale	Number of questions used to derive sub-scale score	NEI-VFQ question numbers used	Questions asked
General health	1	1	Perception of overall health (5 levels)
General vision	1	2	Perception of eyesight (6 levels)
Ocular pain	2	4,19	Pain and discomfort in and around the eyes and the degree of ocular pain
Near activities	3	5,6,7	Difficulty reading ordinary print in newspapers, performing work or hobbies requiring near vision, and finding something on a crowded shelf
Distance activities	3	8,9,14	Difficulty reading street signs or names of shops, going down steps, stairs or kerbs in poor light and visiting people in unfamiliar surroundings
Vision specific:			
Social functioning	2	11,13	Difficulty seeing how people react and visiting people in unfamiliar surroundings
Mental health	4	3,21,22,25	Worries and frustration about eyesight, embarrassment and loss of control caused by eyesight
Role difficulties	2	17,18	Lack of accomplishment and limitations caused by eyesight
Dependency	3	20,23,24	Need to stay at home, reliance on others, and need of help
Driving	3	15c, 16	Difficulty driving during the daytime and at night
Colour vision	1	12	Difficulty picking out and matching clothes
Peripheral vision	1	10	Difficulty noticing objects off to the side whilst walking
Total VFQ score		All	A total score averaged for the subscale scores listed above

In all analyses visual impairment was defined as a visual acuity equivalent to a Snellen acuity below 6/18 in the better eye (equivalent to the ICD-10 definition category 1 to 5) [17]; reduced visual acuity below 6/12 in the better eye was also considered. Both better eye and binocular acuity (acuity measured with both eyes open) were considered; using better eye acuity allowed the influence of differences in acuity between the eyes to be examined. Causes of visual impairment were not routinely collected. The relevant local research ethics committees of the participating practices approved the study.

### Statistical methods

Statistical analysis was performed using STATA (version 7) taking account of the clustered design of the study (i.e. general practice). All analyses were adjusted for age, gender, and general practice (fitted as a fixed effect to allow for between practice consulting behaviour and for geographical location). Logistic regression was used to examine whether the odds of visual impairment were related to age, gender, socioeconomic status, and NEI-VFQ scores. Age was divided into 3 groups (75 to <80, 80 to 85 and >85), Carstairs deprivation index into quartiles (a missing category of Carstairs deprivation index was also included). With the exception of general health, the majority of respondents reported 'no problems' (that is scores of 100) in the NEI-VFQ subscales (or good to excellent eyesight for general vision) and in the analyses the NEI-VFQ subscales were grouped into those reporting 'problems' versus 'no problems'. Self reported general health and the Total VFQ score were divided into quartiles (using the XTILE command in STATA). For the general health domain, this resulted in the responses 'excellent' and 'very good' being combined (as there were few responses to excellent health), to give a group defined as having no problems. The remaining responses were defined as having poor or moderate health or having few problems with general heath. Odds ratios comparing the baseline group reporting no problems with the remainder being split into 3 approximately equal sized groups were calculated. The independent influence of sub-scale scores on visual impairment was examined after adjustment for other scores (except driving due to the small proportion of drivers).

We also quantified change in NEI-VFQ scores and the proportion of the variability (R^2 ^values) in scores, attributed to age, gender, general practice, logMAR acuity in the better eye (as a categorical variable), binocular status (monocular or binocular) and difference in acuity between the eyes (as a continuous variable) using linear regression models. To normalise heavily skewed VFQ scores, an appropriate transformation was achieved by log transformation after adding a constant (using the LNSKEW command in STATA). This was only possible for general vision, near activities, and the total VFQ score. A similar procedure has been described elsewhere [8].

## Results

In total, 4340 older people from the 20 practices had participated in the initial health screen between 1995 and 1999. Three to five years later 1465 participants had died, 252 had moved away and 34 could not be traced. Of the 2875 surviving participants, 2589 (90%) were invited to a vision test, 1807 participated (70%), 670 refused (26%) and 112 (4%) were too ill. The mean age of participants was 83 years (77 to101 years); and the majority were female (64%). Complete data, including a measure of visual acuity and a completed NEI-VFQ, were available for 1785 participants. The prevalence of reduced visual acuity less than 6/12 in the better eye was 33% (n = 585/1785), and 14% (n = 243/1785) for visual impairment (defined as visual acuity less than 6/18 in the better eye). The cause of visual impairment was only available for an unrepresentative minority of participants, and is not considered further.

Table 2 shows the influence of age, gender, social economic status, NEI-VFQ sub-scale and total scores on visual impairment. The likelihood of visual impairment increased sharply with age. Females were more likely than males to be visually impaired even after adjustment for age. There was a weak association with Carstairs Index with those living in less privileged areas being less likely to have visual impairment; the association between Carstairs index and reduced visual acuity was similar but appeared stronger. Of the NEI-VFQ sub-scales, self-reported general health was moderately associated with visual impairment and there was no association with ocular pain. Reporting of problems in the remaining VFQ sub-scales and total scores were strongly related to visual impairment. In a subgroup that responded to questions concerning driving, visual impairment was strongly related to increased difficulties with driving. Associations with worst eye as well as better eye acuity were also examined; in general, associations were similar but weaker (data not presented).

**Table 2:** Association between age, gender, social status, NEI-VFQ sub-scale scores and visual impairment

**Exposure**	**N (%)**	**Number with VA<6/12 (%)**	**OR VA<6/12 (95% CI)**	**Number with VA<6/18 (%)**	**OR VA<6/18 (95% CI)**
**Age (years)**					
75 to <80	310 (17)	56 (18)	1.00	17 (5)	1.00
80 to 85	881 (49)	226 (26)	1.31 (0.93, 1.85)	83 (9)	1.68 (0.97, 2.93)
>85	594 (33)	303 (51)	4.44 (3.14, 6.29)	143 (24)	5.22 (3.05, 8.95)
Total	1785	585 (33)		243 (14)	
P-value †			<0.001		<0.001
**Gender**					
Male	650 (36)	174 (27)	1.00	61 (8)	1.00
Female	1135 (64)	411 (36)	1.31 (1.05, 1.65)	182 (16)	1.57 (1.13, 2.16)
Total	1785	585 (33)		243 (14)	
P-value			0.019		0.006
**Carstairs deprivation index**					
High (privileged)	388 (22)	149 (38)	1.00	61 (16)	1.00
Higher middle	386 (22)	140 (36)	0.92 (0.65, 1.31)	59 (15)	1.05 (0.67, 1.64)
Lower middle	389 (22)	124 (32)	0.68 (0.47, 1.00)	57 (15)	0.92 (0.56, 1.50)
Low (less privileged)	403 (23)	115 (29)	0.69 (0.46, 1.04)	40 (10)	0.72 (0.41, 1.26)
Missing	219 (12)	57 (26)	0.56 (0.37, 0.85)	26 (12)	0.79 (0.46, 1.36)
Total	1785	585 (33)		243 (14)	
P-value †			0.002		0.167
**General health**					
No problems	142 (8)	41 (29)	1.00	16 (11)	1.00
Few	493 (28)	129 (26)	0.78 (0.50, 1.22)	44 (9)	0.68 (0.36, 1.29)
Moderate health	614 (34)	198 (32)	1.03 (0.67, 1.59)	91 (15)	1.16 (0.64, 2.11)
Poor health	534 (30)	216 (40)	1.43 (0.93, 2.22)	91 (17)	1.25 (0.68, 2.28)
Total	1783	584 (33)		242 (14)	
P-value †			<0.001		0.018
**General vision**					
No problems	1206 (68)	244 (20)	1.00	50 (4)	1.00
Problems	577 (32)	339 (59)	5.75 (4.53, 7.31)	192 (33)	11.0 (7.72, 15.5)
Total	1783	583 (33)		242 (14)	
P-value			<0.001		<0.001
**Ocular pain**					
No problems	982 (55)	316 (32)	1.00	120 (12)	1.00
Problems	801 (45)	267 (33)	1.04 (0.83, 1.29)	122 (15)	1.22 (0.91, 1.64)
Total	1783	583 (33)		242 (14)	
P-value			0.751		0.176
**Near activities**					
No problems	838 (47)	156 (19)	1.00	27 (3)	1.00
Problems	940 (53)	422 (45)	3.48 (2.75, 4.40)	213 (23)	8.35 (5.44, 12.8)
Total	1778	540 (31)		240 (14)	
P-value			<0.001		<0.001
**Distance activities**					
No problems	839 (50)	185 (22)	1.00	40 (5)	1.00
Problems	856 (51)	339 (40)	2.11 (1.67, 2.66)	178 (21)	4.91 (3.37, 7.14)
Total	1695 ‡	524 (31)		218 (13)	
P-value			<0.001		<0.001
**Social function**					
No problems	1420 (80)	357 (28)	1.00	95 (7)	1.00
Problems	355 (20)	219 (62)	4.71 (3.60, 6.16)	145 (41)	9.38 (6.81, 12.9)
Total	1775	576 (32)		240 (14)	
P-value			<0.001		<0.001
**Mental health**					
No problems	954 (54)	218 (23)	1.00	56 (6)	1.00
Problems	829 (46)	365 (44)	2.54 (2.03, 3.17)	186 (22)	4.46 (3.20, 6.23)
Total	1783	583 (33)		242 (14)	
P-value			<0.001		<0.001
**Role difficulties**					
No problems	1058 (60)	246 (23)	1.00	52 (5)	1.00
Problems	721 (41)	33 (46)	2.79 (2.23, 3.49)	188 (26)	6.77 (4.81, 9.55)
Total	1779	579 (33)		240 (13)	
P-value			<0.001		<0.001
**Dependency**					
No problems	1381 (78)	335 (24)	1.00	91 (7)	1.00
Problems	400 (22)	246 (64)	4.80 (3.70, 6.24)	150 (38)	7.45 (5.44, 10.2)
Total	1781	581 (33)		241 (14)	
P-value			<0.001		<0.001
Driving *					
No problems	299 (55)	29 (10)	1.00	3 (1)	1.00
Problems	246 (45)	79 (32)	4.63 (2.81, 7.62)	38 (15)	24.5 (6.57, 91.1)
Total	545	108 (20)		41 (8)	
P-value			<0.001		<0.001
**Colour vision**					
No problems	1578 (90)	445 (28)	1.00	146 (9)	1.00
Problems	178 (10)	121 (68)	5.39 (3.74, 7.78)	89 (38)	9.86 (6.77, 14.3)
Total	1756	566 (32)		235 (13)	
P-value			<0.001		<0.001
**Peripheral vision**					
No problems	1300 (75)	326 (25)	1.00	94 (7)	1.00
Problems	440 (25)	227 (52)	3.02 (2.37, 3.87)	95 (24)	5.03 (3.68, 6.87)
Total	1740	553 (32)		189 (11)	
P-value			<0.001		<0.001
**Total VFQ score**					
No problems	442 (25)	75 (17)	1.00	12 (3)	1.00
Few problems	447 (26)	103 (23)	1.45 (1.02, 2.07)	21 (5)	1.72 (0.82, 3.58)
Moderate vision	456 (26)	136 (30)	2.01 (1.43, 2.83)	41 (9)	3.37 (1.72, 6.60)
Poor vision	438 (25)	269 (61)	6.93 (4.93, 9.75)	168 (38)	19.5 (10.5, 36.5)
Total	1783	583 (33)		242 (14)	
P-value †			<0.001		<0.001

Odds ratios adjusted for age, gender, and general practice throughout (except for age which is adjusted for gender and practice, and gender which is adjusted for age and practice)

† Exposure variable treated as a score. ‡ Slightly lower number of respondents to this domain score, as participants responded that they had stopped going out to see films, plays, or sport events as they were not interested in doing this or due to non eyesight related reasons

*Odds ratios for VA<6/18 based on 463 as 4 practices did not have drivers with VA<6/18

As different sub-scales scores may be inter-related (that is, difficulty with general vision may reflect difficulties with near and/or distance vision) the independent influence of these scores on visual impairment was determined (Table 3). After adjustment for demographic and other sub-scale scores, difficulties with general vision, near activities, and social functioning remained associated with visual impairment. Difficulties with dependency and colour vision were of borderline statistical significance.

**Table 3:** Association between NEI-VFQ sub-scale scores and visual impairment, adjusted for age, gender, practice and other sub-scale scores listed in the table

**Exposure**	**OR VA<6/12 (95% CI)**	**OR VA<6/18 (95% CI)**
**General vision**		
No problems	1.00	1.00
Problems	3.64 (2.68, 4.95)	4.77 (3.03, 7.53)
P-value	<0.001	<0.001
**Near activities**		
No problems	1.00	1.00
Problems	1.62 (1.18, 2.22)	2.32 (1.30, 4.15)
P-value	0.003	0.005
**Distance activities**		
No problems	1.00	1.00
Problems	0.74 (0.54, 1.02)	0.91 (0.54, 1.52)
P-value	0.063	0.709
**Social function**		
No problems	1.00	1.00
Problems	1.84 (1.25, 2.71)	2.52 (1.55, 4.11)
P-value	0.002	<0.001
**Mental health**		
No problems	1.00	1.00
Problems	0.94 (0.67, 1.32)	0.72 (0.42, 1.25)
P-value	0.726	0.250
**Role difficulties**		
No problems	1.00	1.00
Problems	0.85 (0.59, 1.23)	1.22 (0.69, 2.17)
P-value	0.381	0.496
**Dependency**		
No problems	1.00	1.00
Problems	1.74 (1.18, 2.56)	1.73 (1.05, 2.86)
P-value	0.005	0.033
**Colour vision**		
No problems	1.00	1.00
Problems	1.59 (1.00, 2.55)	2.25 (1.35, 3.73)
P-value	0.052	0.002
**Peripheral vision**		
No problems	1.00	1.00
Problems	1.21 (0.86, 1.69)	0.95 (0.59, 1.53)
P-value	0.279	0.850

The independent influence of visual acuity, binocular status, and differences in visual acuity between eyes on log transformed scores for general vision, near activities, and total VFQ scores was examined using linear regression (Table 4). Differences in acuity between eyes were defined as less than or equal to a 0.1 difference in LogMAR acuity (equivalent to a difference in Snellen acuity from 6/9 to 6/12) or greater, and compared to those with equal acuity. Although overall differences in these scores were small (e.g. inter quartile range 83 to 97 for the total score) compared to the possible range of measurement (from 0 to 100), scores were strongly related to differences in visual acuity, binocular or monocular status, and differences in acuity between eyes. To examine the relative contribution of visual acuity, demographic factors, binocularity, and between eye differences in acuity in explaining variation in total VFQ scores, the correlations of determination (R^2 ^values) for different accumulative linear regression models are given in Table 5. The R^2 ^value gives the proportion of the variability in total scores explained by different exposure variables. For instance, visual acuity alone explains 16% of the variability in the total score. The addition of demographic variables, binocular status, and difference in acuity between eyes results in 27% of the variability being explained (an increase of 19%). Hence, although visual acuity alone, only accounts for less than a fifth of the variability, it appears to be the most important determinant of total VFQ score.

**Table 4:** Mean scores (95% CI) by visual acuity (better eye), binocular status, and by between eye differences in visual acuity

**Exposure**	**Number (%)**	**General vision**	**Near activities**	**Total VFQ score**
**Visual acuity (better eye)**				
6/9 or better	877 (50)	79.7 (78.8, 80.5)	99.4 (99.2, 99.5)	94.7 (94.3, 95.1)
<6/9 to 6/12	387 (22)	75.0 (73.6, 76.4)	98.6 (98.1, 98.9)	92.8 (92.0, 93.4)
<6/12 to 6/18	277 (16)	72.1 (70.3, 73.8)	97.2 (96.1, 98.0)	91.2 (90.1, 92.2)
<6/18	203 (12)	60.4 (57.7, 63.0)	88.1 (82.5, 92.0)	83.1 (80.5, 85.3)
P-value †	1744	<0.001	<0.001	<0.001
**Binocular status**				
Binocular	1561 (90)	76.7 (76.0, 77.3)	98.9 (98.7, 99.0)	93.6 (93.3, 93.9)
Monocular ‡	183 (10)	64.7 (62.0, 67.3)	94.7 (92.0, 96.5)	83.1 (80.4, 85.5)
P-value	1744	<0.001	<0.001	<0.001
**Difference in acuity between eyes**				
Equal acuity	185 (12)	80.2 (78.4, 81.9)	99.4 (99.1, 99.6)	95.4 (94.6, 96.0)
≤ 0.1 LogMAR	657 (42)	77.9 (76.9, 78.9)	99.2 (99.0, 99.3)	94.5 (94.0, 94.9)
>0.1 LogMAR	719 (46)	75.4 (74.4, 76.4)	98.4 (98.1, 98.7)	92.6 (92.0, 93.1)
P-value †		<0.001	<0.001	<0.001

Scores by levels of visual acuity, binocular status, and differences in acuity between eyes adjusted for age, gender, practice, and for other exposures listed in the table throughout

‡ 183 participants could not read the chart at 1 metre in one eye

**Table 5:** Variance in total domain scores explained by visual acuity (better eye), demographic variables, binocular status, and difference in acuity between eyes

**Exposure**	**R^2 ^for VFQ Total score**
Visual acuity	0.16
Age, gender, practice	0.08
Visual acuity, age, gender, practice	0.20
Visual acuity, age, gender, practice, binocular status	0.25
Visual acuity, age, gender, practice, binocular status, difference in acuity between eyes	0.27

## Discussion

In this older population, self-reported difficulties with general vision, near activities, colour vision, vision related social functioning, and dependency on others to perform visual tasks were associated independently with reduced visual acuity and visual impairment. These findings are in agreement with other studies where loss of vision has been found to be associated with increased social isolation, depression [11,18], and restriction of daily activities [19]. Reported difficulties with colour vision may reflect the increased likelihood of cataract within this elderly population [20]. These important sub-scale scores can be used to give an independent measure of visual difficulty amongst the elderly. Concerns have been raised about the validity of the NEI-VFQ in a general population, since it was developed primarily amongst those with eye disease, and scores tend to be high and with a limited range of measurement in the general population [12]. The general population used in this study was elderly where visual problems are more prevalent, and a substantial proportion of participants reported difficulties, especially with certain sub-scales. For example, over half reported difficulties with either distance or near activities, and around 40% reported difficulties with role difficulties, mental health and driving, and a quarter problems with peripheral vision. The NEI-VFQ is scored by summing the ranks of responses to certain questions for different domain scores (see Table 1), resulting in a Likert scale [21]. A similar approach is used for other visual function questionnaires [21-23]. This approach assumes that the response to each question are of similar importance, and that each category of response represents an equal interval along a continuous dimension from 'no problems' to 'problems'. However, the intervals at the 'ceiling' and 'floor' of this scale are unbounded, which some argue invalidates the Likert scale derived [21,23]. In addition, allowing participants not to answer certain questions as being no longer relevant (such as the questions used for distance activities, see Table 2), may invalidate the measure further. Despite these caveats we have shown strong associations between certain NEI-VFQ scores and cut-offs in visual acuity describing visual impairment and reduced visual acuity, suggesting that the everyday problems experienced in vision related activities are of considerable concern in this age group.

Visual acuity alone, although showing strong associations with NEI-VFQ scores and useful in population vision testing, is a relatively limited measure of vision performance – only explaining up to a fifth of the variation in total VFQ score (comparable to levels reported in other studies of populations with eye disease e.g. age related macular degeneration and glaucoma) [6,7]. Adding other factors such as demographic variables (age, gender, and the general practice where subjects were examined), binocular status, and difference in acuity between eyes led to just over a quarter of the variation in total VFQ score being explained. Measures of deprivation were not related to self reported vision function. Hence, although visual acuity alone appears to be the most important determinant of the VFQ score, it only gives a limited measure of visual performance. It is possible that inclusion of a broader spectrum of tests, such as contrast sensitivity, colour vision and assessment of the visual field may provide a more comprehensive picture of visual performance. In addition, there will be a range of other social and psychological factors that influence a person's response to reduced vision. Alas, we collected only limited data on other possible influencing variables such as depression, social circumstances or personality at the follow up visit, as this was not the main focus of our study. Other studies support the conjecture that other measures besides visual acuity are associated with an individuals perception of visual performance [1,24,25].

Although this study showed that self-reported problems on the NEI-VFQ are associated with visual impairment, problems with some domain scores were more strongly associated with visual impairment (such as near activities, distance activities and general vision) than other domain scores (e.g. ocular pain and colour vision). On the basis of the total VFQ score categories (see Table 2) taking those classified with poor vision (in the lowest quartile of the VFQ score) as screen positive and the rest as screen negative, would result in a detection rate of visual impairment of 69% (168/242) and a false positive rate of 18% (270/1541). Thus approximately 1 in 5 people without visual impairment would be screen positive using this definition. The question referring to driving was more sensitive (93% = 38/41) but had a higher false positive rate (52% = 261/504), and was only answered by 30% of the sample.

## Conclusion

We conclude that the NEI-VFQ appears to be a useful instrument for measuring visual difficulties that can be used in studies of elderly people with significant levels of visual impairment. The NEI-VFQ could be usefully employed when it is logistically or financially impossible to examine all subjects [26]. The VFQ measures important difficulties that are strongly related to definitions of visual impairment (based on established cut-offs in visual acuity), which might be used as an additional measure of visual ability.

## Abbreviations

National Eye Institute Visual Function Questionnaire (NEI-VFQ)

## Sources of support

Royal National Institute for the Blind (RNIB) and the Medical Research Council, UK.

## Competing interests

The author(s) declare that they have no competing interests

## Authors' contributions

All authors contributed to the formulation of the hypothesis. CGO and ARR performed the statistical analysis. CGO drafted the manuscript to which all authors contributed, and is guarantor. All authors read and approved the final manuscript.

## Pre-publication history

The pre-publication history for this paper can be accessed here:


